# Validating the Depression Anxiety Stress Scales‐21 in Community Mothers and Fathers Across the Infant's First Year

**DOI:** 10.1111/cch.70099

**Published:** 2025-06-05

**Authors:** Birgitta Kerstis, Peter Jönsson, Mariette Derwig, Kent W. Nilsson, Inger Kristensson Hallström, Sara Lindeberg

**Affiliations:** ^1^ Division of Caring Sciences School of Health, Care and Social Welfare, Mälardalen University Västerås Sweden; ^2^ Department of Psychology Faculty of Education, Kristianstad University Kristianstad Sweden; ^3^ Child and Family Health, Department of Health Sciences Faculty of Medicine, Lund University Lund Sweden; ^4^ Center for Clinical Research Central Hospital of Västerås, Uppsala University Uppsala Sweden; ^5^ Division of Public Health Sciences School of Health, Care and Social Welfare, Mälardalen University Västerås Sweden; ^6^ Lund Clinical Research on Externalizing and Developmental psychopathology (LU‐CRED), Department of Clinical Sciences Faculty of Medicine, Lund University Lund Sweden

**Keywords:** Consensus‐based Standards for the Selection of Health Status Measurement Instruments (COSMIN), Depression Anxiety Stress Scales‐21 (DASS‐21), Edinburgh Postnatal Depression Scale (EPDS), fathers, mothers, postnatal parental distress, validity

## Abstract

**Background:**

To investigate the effects of different types of parental health status on child development and health, valid parental distress measurement instruments are needed. The aim was to assess the psychometric measurement properties of the 21‐item Depression Anxiety Stress Scales (DASS‐21) used during the postnatal year in community samples of Swedish mothers and fathers.

**Methods:**

Data were collected at postnatal months 1, 6 and 12 from 66 mothers (mean age 31 years) and 58 fathers (mean age 32 years). Psychometric measurement properties were assessed with interitem analysis and intra‐individual measurements cross‐correlations. As part of assessing construct validity, the hypotheses that the levels of the different types of postnatal distress as measured by the DASS‐21 would be higher in mothers than in fathers were tested.

**Results:**

The DASS‐21 internal consistencies were good for both parent groups. The DASS‐21 depression scale correlated strongly with the Edinburgh Postnatal Depression Scale (EPDS) in mothers and moderately strongly in fathers at each timepoint. Correlations between the DASS‐21 anxiety scale and the EPDS ‘anxiety component’ were mostly in the low‐to‐moderate range. The DASS‐21 stress scale significantly distinguished parental distress levels in the expected direction, as did the total DASS‐21 scale.

**Conclusions:**

Similar DASS‐21 psychometric properties to those demonstrated for non‐perinatal adult populations were indicated. The results suggest the usefulness of the DASS‐21 during the early, middle and late postnatal periods for measuring types of as well as general maternal and paternal distress. Further analysis in larger perinatal parental samples for more conclusive results is warranted.

## Key Messages

- This is the first study to assess the validity of the Depression Anxiety Stress Scales‐21 (DASS‐21) in a perinatal parental sample including both mothers and fathers.
- The results were in line with those previously demonstrated for nonperinatal adult populations, thus suggesting the usefulness of the DASS‐21 during the early, middle and late postnatal periods for measuring types of, as well as general, maternal and paternal distress.
- The validities of the DASS depression (DASS‐D) and DASS anxiety (DASS‐A) scales seem comparable to those of other instruments currently used for assessing the constructs of perinatal parental depressive symptoms and anxiety; an advantage of using the DASS‐21 to assess types of perinatal parental distress is its inclusion of a stress measure, the DASS stress scale (DASS‐S).

## 1 Introduction

Perinatal distress is one or more states of pre‐ or postnatal parental depressive symptomatology, anxiety, or stress, including experiencing stressful life events from conception until the child is 1 year old (Ross et al. 2020). From a life course perspective, the effects of maternal perinatal, particularly postnatal, depressive symptoms on the offspring are well‐established, including impacts on the child's sensitivity to maternal signals, sleeping patterns, and motor, cognitive, language, emotional, social and behavioural development (Meaney 2018; Slomian et al. 2019; Rogers et al. 2020). Accordingly, screening for perinatal maternal depression is standard in many high‐income countries (El‐Den et al. 2022).

In contrast, the effects of perinatal distress in the other parent, including depressive symptoms, on the child have received less attention (Challacombe et al. 2023) and perinatal depression screening in fathers or other non‐birthing parents is not the norm (Hunt et al. 2022). However, research on paternal postnatal depressive symptoms is accumulating (Glasser and Lerner‐Geva 2019; Berg et al. 2022; Field 2018a; Cameron et al. 2016; Paulson and Bazemore 2010). Depressive symptoms rates in fathers are generally lower compared with those in mothers (Glasser and Lerner‐Geva 2019). Paternal depressive symptoms have been demonstrated to increase child risks in terms of impairments in parenting capacity (Paulson et al. 2009; Davis et al. 2011), as well as long‐term psychopathology and functioning in the child, independently of maternal postnatal depression (Ramchandani et al. 2008).

The validity of depressive symptoms measurements in mothers is well‐established (Sambrook Smith et al. 2022), whereas measuring paternal depressive symptoms has methodological limitations (Field 2018a). For instance, the addition of anger assessments to the more traditional depressive symptoms in studies of fathers suggests a need to include gender‐specific items in postnatal depressive symptoms measures (Psouni et al. 2017; Macdonald et al. 2020). Nevertheless, the construct of paternal postnatal depression is currently measured exclusively with instruments developed or used for assessing maternal depression (Berg et al. 2022).

Maternal and paternal depressive symptoms have been repeatedly weakly associated (Kiviruusu et al. 2020; Kerstis et al. 2016; Paulson et al. 2016), and depressive symptoms in early parenthood predict later couple separation (Widarsson et al. 2019). A recent meta‐analysis found that postnatal depression occurred in both parents, with 2.4% and 3.2% of parental dyads affected during the early and late postnatal periods, respectively (Smythe et al. 2022). It would thus be important to consider both parents, including the potential interactions between their respective mental health conditions, when studying the effects of parental distress on child development and health (Challacombe et al. 2023; Pietikäinen et al. 2020; Möller et al. 2015).

The well‐studied impact of parental depressive symptoms on the child's health may also be influenced or confounded by other types of parental distress (or vice versa) known to be associated with depressive symptoms, including anxiety and stress (Field 2018b; Deltsidou et al. 2018; Skjothaug et al. 2018). Therefore, it is also appropriate to include distress assessments beyond those for depression in studies assessing the child effects of perinatal parental health. The need for such studies is increasingly highlighted (Ross et al. 2020; Rogers et al. 2020; Sinesi et al. 2019), as is the necessity to scrutinize the psychometric properties of distress measurement instruments used during the perinatal period (Berg et al. 2022; Adhikari et al. 2021). For example, studies on the effects of postnatal anxiety in either mothers or fathers on their offspring increasingly suggest its unique impacts, distinct from those of depressive symptoms (Möller et al. 2015; Field 2018b; Sweeney and Wilson 2023). However, the findings have been inconsistent (Sambrook Smith et al. 2022; Sweeney and Wilson 2023; Meades and Ayers 2011). Consequently, the inclusion of anxiety screening tools in community‐based maternal and child health settings has been rare (Bhat et al. 2022), and greater consensus regarding anxiety assessment methodology is warranted.

The transition to parenthood can also be stressful, and perinatal stress in mothers and fathers has child development and health implications (Challacombe et al. 2023; Gao et al. 2022). Interestingly, one paternal distress study showed that neither depression nor anxiety symptoms were related to child outcomes after adjusting for paternal stress (Challacombe et al. 2023), which emphasizes the importance of including different distress assessments in studies on child effects of parental health conditions. Consistent with study results concerning levels of depressive symptoms and anxiety (Glasser and Lerner‐Geva 2019; Hantsoo and Epperson 2017; Nguyen et al. 2023), studies demonstrate higher perinatal stress levels in mothers than in fathers (Challacombe et al. 2023; Hildingsson and Thomas 2014), leading to maternal distress in general appearing more prevalent compared to paternal distress.

The Depression Anxiety Stress Scales (DASS) includes three self‐reported scales designed to measure the emotional states of depression, anxiety and stress in adults (Lovibond and Lovibond 1995). The widely used 21‐item DASS (DASS‐21) (Antony et al. 1998) is a shorter version of the original 42‐item DASS, translated to at least 44 different languages (Lee et al. 2019). Lee and colleagues (Lee et al. 2019) concluded in their systematic review of the DASS‐21 that its psychometric robustness indicates that it can be used to assess the negative emotions of depression, anxiety and stress, as well as general psychological distress in both general and clinical adult populations. These review results were in line with the results from a study validating the Swedish version of the DASS‐21 in student, primary care and psychotherapy population samples (Alfonsson et al. 2017).

The DASS‐21 was thus not developed specifically for the perinatal period. It has occasionally been used in maternal populations (Shahmi Ruslan et al. 2022), and it is acknowledged to measure distress types that are contextually appropriate for the perinatal period (Meades and Ayers 2011; Miller et al. 2006; Evans et al. 2015) but has not yet been validated for this purpose.

The study aim was to assess the psychometric measurement properties of the DASS‐21 in Swedish community samples of mothers and fathers at three postnatal timepoints (infant ages 1 month, 6 months and 12 months respectively). The well‐established Edinburgh Postnatal Depression Scale (EPDS) (Sambrook Smith et al. 2022; Cox et al. 1987) was used as a comparator instrument. The three‐item ‘anxiety component’ of the EPDS (EPDS‐3A) has been suggested to be used to screen for anxiety disorders in mothers and fathers (Matthey 2008), and it was included in the analysis as a comparator measurement of anxiety. Guided by the Consensus‐based Standards for the Selection of Health Status Measurement Instruments (COSMIN) framework (Terwee et al. 2007; Mokkink et al. 2010a; Mokkink et al. 2010b), scale internal consistency and construct validity were assessed. The latter included convergent and discriminant validity and testing of the hypotheses that the levels of postnatal depressive symptoms, anxiety and stress, as well as general distress as measured by the total DASS‐21, would be higher in mothers than in fathers.

## 2 Methods

### 2.1 Samples Recruitment and Data Collection

This study analysed the parental survey data from the second, third and fourth waves of the longitudinal Scania Birth Cohort pilot study – Health development in a life course perspective (Lindeberg et al. 2024). The recruitment of parents, which could include single mothers with sole custody as well as parental dyads that were not couples, took place at an antenatal clinic during the third pregnancy trimester. Multiple pregnancy, donor egg pregnancy, insufficient skills in the Swedish language and premature births were exclusion criteria. The children were after birth enrolled in postnatal care in Scania, the most southern region of Sweden, following the standard no‐cost national program for child health services (CHS). Data were collected between May 2019 and June 2021 at CHS visits when the infant was 4–6 weeks (1 month), 6 months and 12 months, herein referred to as times 1, 2 and 3 (T1–T3), respectively. The study participants for the present study were the 66 mothers and 58 fathers who completed at least one of the surveys at the three data collection timepoints for this study. Fifty‐seven mothers and fathers from each parent group (86.1% and 98.3%, respectively) were living together as couples, and one participating parental dyad was not a couple. The parental surveys were completed at home, and the parents were encouraged to do so separately. The characteristics of the two parent groups are described in Table 1. Due to unforeseen obstacles including prolonged restrictions from the COVID‐19 pandemic, all child health centre data collection was terminated in June 2021. This is the main reason for the declining numbers of participants from T1 to T3. Two mothers and two fathers terminated their follow‐up study participation of their own accord. See Table 2 for the numbers of mothers and fathers participating at the different data collection timepoints (T1–T3).

**TABLE 1:** Descriptive characteristics of the sample.

	**Mothers** *N* = 66	**Fathers** *N* = 58
**Parental age**, years mean (SD)	31.2 (4.0)	32.3 (5.6)
**Infant age** ^a^, months mean (SD)		
T1, 1 month	1.6 (0.6)	1.7 (0.6)
T2, 6 months	6.3 (0.5)	6.5 (0.8)
T3, 12 months	12.0 (0.6)	12.1 (0.5)
**Highest education**, *n* (%)		
Compulsory school (9 years)	2 (3.0%)	3 (5.2%)
Senior high school	19 (28.8%)	21 (36.2%)
University (≤ 3 years)	21 (31.8%)	15 (25.9%)
University (> 3 years)	24 (36.4%)	14 (24.1%)
Post‐graduate education	0	0
*Information missing*	0	5 (8.6%)
**Cohabitation status**, *n* (%)		
Living with the other parent	61 (92.4%)	57 (98.3%)
Living alone	3 (4.5%)	0
Living with their own parents	2 (3.0%)	1 (1.7%)
**Region of birth**, *n* (%)		
Sweden	60 (90.9%)	52 (89.7%)
Scandinavia (not Sweden)	1 (1.5%)	0
Europe (not Scandinavia)	3 (4.5%)	3 (5.2%)
Outside of Europe	2 (3.0%)	2 (3.4%)
*Information missing*	0	1 (1.7%)

*Note:* T1–T3, infant age at the child health services visit = postnatal data collection timepoints.

^a^ Infant age when the parents completed the T1–T3 surveys, respectively.

**TABLE 2:** Measurement internal reliability coefficients, scale missing data, means and standard deviations (SD), and percentages of lowest possible score in mothers and fathers at the three study timepoints, T1–T3.

**Postnatal time** (infant age)	**T1** (1 month)				**T2** (6 months)				**T3** (12 months)			
	**Mothers**		**Fathers**		**Mothers**		**Fathers**		**Mothers**		**Fathers**	
No. of participants, *N* ^a^	*N* = 62		*N* = 55		*N* = 57		*N* = 50		*N* = 36		*N* = 34	
	ω	α	ω	α	ω	α	ω	α	ω	α	ω	α
**DASS‐D** internal reliability coefficients	**0.91**	**0.88**	**0.84**	**0.77**	**0.85**	**0.83**	**0.91**	**0.87**	**0.90**	**0.88**	**0.87**	**0.77**
No. of responding, *n* ^b^; % scale missing data	*n* = 61; 1.6%		*n* = 51; 7.3%		*n* = 54; 5.3%		*n* = 48; 4.0%		*n* = 35; 2.8%		*n* = 33; 2.9%	
Mean (SD)	1.97 (3.15)		1.39 (2.30)		1.85 (2.71)		1.40 (2.45)		2.00 (3.24)		1.06 (1.75)	
Percentage lowest possible score	36.1%		47.1%		37.0%		52.1%		45.7%		54.5%	
**DASS‐A** internal reliability coefficients	**0.82**	**0.76**	**0.79**	**0.74**	**0.79**	**0.75**	**0.85**	**0.81**	**0.84**	**0.84**	**0.93**	**0.80**
No. of responding, *n* ^b^; % scale missing data	*n* = 62; 0%		*n* = 49; 10.9%		*n* = 54; 5.3%		*n* = 48; 4.0%		*n* = 36; 0%		*n* = 33; 2.9%	
Mean (SD)	1.35 (2.43)		0.82 (1.82)		1.20 (2.13)		0.92 (2.17)		1.42 (2.79)		0.85 (1.86)	
Percentage lowest possible score	53.2%		67.3%		53.7%		66.7%		50.0%		66.7%	
**DASS‐S** internal reliability coefficients	**0.84**	**0.83**	**0.83**	**0.67**	**0.87**	**0.87**	**0.89**	**0.89**	**0.94**	**0.93**	**0.85**	**0.83**
No. of responding, *n* ^b^; % scale missing data	*n* = 59; 4.8%		*n* = 51; 7.3%		*n* = 54; 5.3%		*n* = 49; 2.0%		*n* = 36; 0%		*n* = 33; 2.9%	
Mean (SD)	4.47 (3.92)		2.59 (3.16)		4.57 (4.04)		3.08 (3.55)		4.53 (5.02)		2.76 (2.92)	
Percentage lowest possible score	18.6%		37.3%		16.7%		32.7%		22.2%		30.3%	
**DASS‐Tot** internal reliability coefficients	**0.94**	**0.93**	**0.92**	**0.86**	**0.93**	**0.92**	**0.95**	**0.94**	**0.95**	**0.95**	**0.94**	**0.91**
No. of responding, *n* ^b^; % scale missing data	*n* = 59; 4.8%		*n* = 49; 10.9%		*n* = 54; 5.3%		*n* = 47; 6.0%		*n* = 35; 2.8%		*n* = 33; 2.9%	
Mean (SD)	7.85 (8.74)		4.63 (6.26)		7.63 (8.00)		5.55 (7.51)		7.60 (9.76)		4.67 (5.81)	
Percentage lowest possible score	13.6%		26.0%		13.0%		27.1%		14.3%		17.6%	
**EPDS** internal reliability coefficients	**0.89**	**0.88**	**0.86**	**0.83**	**0.87**	**0.86**	**0.86**	**0.84**	**0.89** ^c^	**0.88** ^c^	**0.83** ^c^	**0.79** ^c^
No. of responding, *n* ^b^; % scale missing data	*n* = 61; 1.6%		*n* = 53; 3.6%		*n* = 57; 0%		*n* = 48; 4.0%		*n* = 35; 2.8%		*n* = 33; 2.9%	
Mean (SD)	6.54 (5.24)		4.19 (4.07)		5.98 (4.81)		3.92 (3.92)		5.69 (5.12)		2.70 (2.87)	
Percentage lowest possible score	11.5%		15.1%		10.5%		18.8%		8.6%		21.2%	
**EPDS‐3A** internal reliability coefficients	**0.76**	**0.73**	**0.72**	**0.70**	**0.76**	**0.72**	**0.78**	**0.77**	**0.75**	**0.74**	**0.80**	**0.68**
No. of responding, *n* ^b^; % scale missing data	*n* = 61; 1.6%		*n* = 55; 0%		*n* = 57; 0%		*n* = 49; 2.0%		*n* = 36; 0%		*n* = 33; 2.9%	
Mean (SD)	2.66 (2.07)		1.67 (1.76)		2.33 (2.05)		1.69 (1.85)		2.58 (2.23)		1.18(1.42)	
Percentage lowest possible score	14.8%		34.5%		21.1%		30.6%		16.7%		39.4%	

Higher scores on DASS‐D, DASS‐A, DASS‐S, EPDS and EPDS‐3A represent higher risk of distress.

Abbreviations: α, Cronbach's alpha coefficient; ω, McDonald's omega coefficient; DASS, The Depression Anxiety Stress Scales‐21; DASS‐A, The DASS‐21 Anxiety scale (7 items); DASS‐D, The DASS‐21 Depression scale (7 items); DASS‐S, The DASS‐21 Stress scale (7 items); DASS‐Tot, The DASS‐21 Total scale (21 items); EPDS, The Edinburgh Postnatal Depression Scale (10 items); EPDS‐3A, The EPDS ‘anxiety component’ (3 items).

^a^ No. of participants who filled in the questionnaire at each timepoint.

^b^ No. of respondents with complete measurement data.

^c^ Coefficient based on EPDS items 1–9, due to zero variance for the EPDS item no. 10 (all parents scored zero).

### 2.2 Measures

#### 2.2.1 Depression Anxiety Stress Scales‐21

The DASS‐21 is a 21‐item self‐report questionnaire scored on a 4‐point Likert scale (0–3), with three subscales representing depression (DASS‐D), anxiety (DASS‐A) and stress (DASS‐S). DASS‐D focuses on low mood, motivation and self‐esteem, while DASS‐A focuses on physiological arousal, perceived panic and fear, and DASS‐S focuses on tension and irritability (Lovibond and Lovibond 1995; Antony et al. 1998). Each 7‐item subscale has a possible sum score of 21 and the full 21‐item DASS has a total possible score of 66, with higher scores indicating higher distress. The Swedish version of the DASS‐21 (Alfonsson et al. 2017) was used.

#### 2.2.2 Edinburgh Postnatal Depression Scale

The EPDS (Cox et al. 1987) is a 10‐item self‐report questionnaire scored on a 4‐point Likert scale (0–3) with a possible total score of 30. Higher scores indicate more severe depressive symptoms. The EPDS was developed to screen new mothers for depressive symptoms and has been extensively validated for perinatal use (Sambrook Smith et al. 2022). According to a recent scoping review (Berg et al. 2022), the EPDS is currently also the best option for paternal depression screening. The Swedish version of the EPDS (Lundh and Gyllang 1993) was used herein.

Factor analytic studies on data from perinatal maternal populations have frequently found the EPDS to be multidimensional, and most commonly containing a factor with the EPDS items 3, 4 and 5 (Alberto Lopez‐Janer et al. 2021). This factor has been interpreted as an EPDS ‘anxiety component’ known as EPDS‐3A. There is some empirical support for using the EPDS‐3A for perinatal anxiety screening (Matthey et al. 2013; Austin et al. 2021), but its validity or additional value for this purpose as compared with the full 10‐item EPDS has not been established (Fairbrother et al. 2019; Loyal et al. 2020; Smith‐Nielsen et al. 2021). Although this factor structure has not been confirmed in fathers, cut‐offs of the EPDS‐3A for the detection of anxiety disorders have been defined for both mothers and fathers (Matthey 2008).

### 2.3 Statistical Analysis

The analyses were guided by the COSMIN framework, which is an initiative to improve and standardize the development and selection of health measurements (Terwee et al. 2007; Mokkink et al. 2010a; Mokkink et al. 2010b). To assess score interpretability (Terwee et al. 2007), means and standard deviations, percentages of total score missing data, and lowest possible score were calculated for each measurement at each timepoint. Psychometric measurement properties assessed were internal consistency and construct validity. Internal consistency was assessed in interitem analysis using both the McDonald's omega and Cronbach's alpha coefficient methods (Zinbarg et al. 2005). The former is a robust alternative for assessing the internal consistency of ordinal scales (McNeish 2018). Spearman's rank correlation test was used for measurement score correlations. The Wilcoxon signed‐rank test was used to test the hypotheses that mothers' median distress scores would be higher than those of fathers (directional hypotheses), and to calculate effect sizes (*r*) (Field 2013). Statistical analyses were performed using SPSS Statistics (version 28.0; IBM Corp., Armonk, New York, United States) and *jamovi* (version 2.3) computer software retrieved from https://www.jamovi.org.

## 3 Results

### 3.1 Descriptive Statistics Related to Score Interpretability

Missing total scores (Eekhout et al. 2012) were mostly < 5% in both parent groups and not more than 11% at any timepoint. Lowest possible scores were > 15% demonstrating floor effects (Terwee et al. 2007) for the DASS‐D, DASS‐A and DASS‐S in both parent groups, and for the total DASS‐21 in the fathers (Table 2).

### 3.2 Measurement Properties

#### 3.2.1 Internal Consistency

Internal reliability coefficients for the DASS‐21 measurements were high at each timepoint in both parent groups. The DASS‐D omega coefficients ranged from α = 84 to α = 91, comparable to those for the EPDS (α = 0.83 to α = 0.89). The DASS‐A omega coefficients ranged from α = 0.78 to α = 0.93, which are higher values than those demonstrated here for the EPDS‐3A (α = 0.72 to α = 0.80). The DASS‐S and DASS‐21 total score omega coefficients ranged from α = 0.83 to α = 0.94, and α = 0.92 to α = 0.95, respectively (Table 2)**.**

#### 3.2.2 Construct Validity Testing: Convergent and Discriminant Validity

At all three timepoints, the DASS‐D correlated strongly with the EPDS in the mothers (*r*_*s*_ = 0.74–0.77, *p* < 0.001), indicating good convergent validity. These correlations were stronger than those with either anxiety measure (DASS‐A and EPDS‐3A) and usually (i.e., at T1 and T2) stronger than those with the DASS‐S, indicating discriminant validity for the depressive symptoms constructs against the anxiety and stress constructs in the mothers. In the fathers, the DASS‐D correlated moderately strongly with the EPDS at all three timepoints (*r*_*s*_ = 0.63–0.68, *p* < 0.001). These correlations were stronger than those with the two anxiety measures at all timepoints, but less than those with the DASS‐S at T2 and T3. In both parent groups, the DASS‐A and EPDS‐3A correlations were statistically significant only at T1 and T2, when they were low‐to‐moderate (from *r*_*s*_ = 0.41, *p* < 0.05 to *r*_*s*_ = 0.68, *p* < 0.001). In both mothers and fathers, the DASS‐S correlated moderately‐to‐strongly with the two depression measures (*r*_*s*_ = 0.57–0.80, *p* < 0.001), whereas no consistent correlation pattern with the two anxiety measures emerged. At all timepoints in both parent groups, the total DASS‐21 correlated more strongly with the DASS‐S (*r*_*s*_ = 0.89–0.97, *p* < 0.001) than with the two depression measures, DASS‐D and EPDS (*r*_*s*_ = 0.63 to *r*_*s*_ = 0.87, *p* < 0.001), and the two anxiety measures, DASS‐A and EPDS‐3A (*r*_*s*_ = 0.37, *p* < 0.05 to *r*_*s*_ = 0.73, *p* < 0.001) (Table 3).

**TABLE 3:** Parental intra‐individual measurements correlations (Spearman's rank test) at each postnatal timepoint, T1–T3.

	**T1** (1 month) Mothers *N* = 62, Fathers *N* = 55						**T2** (6 months) Mothers *N* = 57, Fathers *N* = 50						**T3** (12 months) Mothers *N* = 36, Fathers *N* = 34					
	**DASS‐D**	**DASS‐A**	**DASS‐S**	**DASS‐Tot**	**EPDS**	**EPDS‐3A**	**DASS‐D**	**DASS‐A**	**DASS‐S**	**DASS‐Tot**	**EPDS**	**EPDS‐3A**	**DASS‐D**	**DASS‐A**	**DASS‐S**	**DASS‐Tot**	**EPDS**	**EPDS‐3A**
**DASS‐D**	—	0.55***	0.58***	0.80***	0.68***	0.55***	—	0.59***	0.73***	0.86***	0.68***	0.61***	—	0.23	0.74***	0.82***	0.63***	0.41**
**DASS‐A**	0.66***	—	0.33*	0.60***	0.52***	0.41**	0.42***	—	0.49***	0.66***	0.68***	0.64***	0.53***	—	0.23	0.43**	0.30*	−0.05
**DASS‐S**	0.57***	0.49***	—	0.89***	0.64***	0.49***	0.66***	0.71***	—	0.96***	0.69***	0.55***	0.80***	0.67***	—	0.94***	0.64***	0.41**
**DASS‐Tot**	0.78***	0.71***	0.92***	—	0.73***	0.54***	0.78***	0.73***	0.97***	—	0.80***	0.68***	0.87***	0.69***	0.97***	—	0.72***	0.37*
**EPDS**	0.75***	0.64***	0.60***	0.75***	—	0.86***	0.74***	0.58***	0.67***	0.74***	—	0.90***	0.77***	0.39*	0.59***	0.63***	—	0.76***
**EPDS‐3A**	0.55***	0.48***	0.60***	0.65***	0.81***	—	0.53***	0.53***	0.64***	0.65***	0.80***	—	0.62***	0.28	0.42**	0.47**	0.84***	—

*Note:* T1–T3 = postnatal data collection timepoints (infant age). Correlation coefficients for the mothers are displayed below the diagonals, and for the fathers above the diagonals.

Abbreviations: DASS, The Depression Anxiety Stress Scales‐21; DASS‐A, The DASS‐21 Anxiety scale (7 items); DASS‐D, The DASS‐21 Depression scale; DASS‐S, The DASS‐21 Stress scale (7 items); DASS‐Tot, The DASS‐21 Total scale (21 items); EPDS, The Edinburgh Postnatal Depression Scale (10 items); EPDS‐3A, The EPDS ‘anxiety component’ (3 items).

*p < 0.05, **p < 0.01, ***p < 0.001.

#### 3.2.3 Construct Validity Testing: Comparisons of Distress Scores Between Parent Groups

Most median distress type levels as measured by the DASS‐S, EPDS and EPDS‐3A were significantly higher in mothers than in fathers with medium effect sizes (from *r* = 0.32, *p* = 0.016 to *r* = 0.43, *p* = 0.008), whereas the differences measured by either the DASS‐D or DASS‐A overall were in the expected direction but not statistically significant. General parental distress levels as measured by the total DASS‐21 were significantly higher in mothers than in fathers at T1 and T2 with small effect sizes (*r* = 0.25, *p* = 0.045 and *r* = 0.25, *p* = 0.049) (Table 4).

**TABLE 4:** Measurement medians (and quartiles) in mothers and fathers in parental dyads at the three study timepoints (T1–T3) and results from Wilcoxon signed‐rank tests of differences between mothers' and fathers' measurement scores with effect sizes (*r*).

	**T1** (1 month) *N* = 54					**T2** (6 months) *N* = 50					**T3** (12 months) *N* = 33				
	Medians (Q1, Q3)					Medians (Q1, Q3)					Medians (Q1, Q3)				
	**Mothers**	**Fathers**	*z*	*p*	*r*	**Mothers**	**Fathers**	*z*	*p*	*r*	**Mothers**	**Fathers**	*z*	*p*	*r*
**DASS‐D**	1 (0, 2)	1 (0, 2)	0.55	0.291	0.08	1 (0, 2)	0 (0, 1.75)	1.08	0.141	0.16	1 (0, 3)	0 (0, 1)	0.92	0.178	0.16
**DASS‐A**	0.5 (0, 2)	0 (0, 1)	1.38	0.084	0.20	0 (0, 2)	0 (0, 1)	0.93	0.176	0.14	1 (0, 2)	0 (0, 1)	1.16	0.124	0.20
**DASS‐S**	3.5 (1.25, 6)	2 (0, 5)	2.45	**0.** **007**	0.35	4 (1, 7)	2 (0, 5)	2.14	**0.** **016**	0.32	3 (1, 8)	2 (0, 4.5)	1.92	**0.** **028**	0.33
**DASS‐Tot**	5.5 (2.25, 10.75)	3 (0, 6.5)	1.70	**0.** **045**	0.25	5 (2, 11)	4 (0, 7)	1.65	**0.** **049**	0.25	4 (1, 14)	3 (1, 7)	1.26	0.105	0.22
**EPDS**	6 (3, 8)	2 (1, 6.75)	2.60	**0.** **005**	0.36	4.5 (2, 9)	3 (1, 6)	2.53	**0.** **001**	0.37	5 (2, 8.5)	2 (1, 3)	2.42	**0.** **008**	0.43
**EPDS‐3A**	2 (1, 4)	1 (0, 3)	2.91	**0.** **002**	0.40	2 (1, 4)	1 (0, 2.5)	1.62	0.053	0.23	2 (1, 4)	1 (0, 2)	2.43	**0.** **008**	0.42

*Note:* T1–T3 = postnatal data collection timepoints (infant age). *N* = number of parental dyads at T1, T2 and T3, respectively. Q1 = quartile 1. Q3 = quartile 3. Higher scores on DASS‐D, DASS‐A, DASS‐S, DASS‐Tot, EPDS and EPDS‐3A represent higher risk of distress. Bold *p* value is statistically significant.

Abbreviations: DASS, The Depression Anxiety Stress Scales‐21; DASS‐A, The DASS‐21 Anxiety scale (7 items); DASS‐D, The DASS‐21 Depression scale (7 items); DASS‐S, The DASS‐21 Stress scale (7 items); DASS‐Tot, The DASS‐21 Total scale (21 items); EPDS, The Edinburgh Postnatal Depression Scale (10 items); EPDS‐3A, The EPDS ‘anxiety component’ (3 items); *z*, standardized test statistic; *p*, *p* value; *r*, effect size.

## 4 Discussion

The psychometric properties of the three DASS‐21 scales as well as the total DASS‐21, used repeatedly across the first (early, middle and late) postnatal year in community samples of mothers and fathers were assessed. Good internal consistencies were demonstrated in both mothers and fathers at all timepoints. The DASS‐D convergent validity was at each timepoint good in the mothers and acceptable in the fathers. DASS‐D discriminant validity was demonstrated against the anxiety and stress constructs for the mothers, and against the anxiety constructs for the fathers. In both mothers and fathers, the DASS‐D measurement properties were superior to those for the DASS‐A. These results indicate that the DASS‐D can be used to identify depressive symptoms in new parents. Further investigation of DASS‐D criterion validity against a gold standard (e.g., structured clinical interview) for depression diagnosis in both mothers and fathers would be desirable.

Few studies have assessed the measurement properties of the DASS‐21 in perinatal parental populations, and to our knowledge, this is the first to include fathers. As part of assessing DASS‐21 construct validity (Mokkink et al. 2010a), it was hypothesized, based on previous study results (Challacombe et al. 2023; Nguyen et al. 2023), that distress levels as measured by each measurement would be higher in mothers than in fathers. The differences were in the expected direction, thus corroborating construct validity, and mostly statistically significant for the total DASS‐21 and the DASS‐S. They were not statistically significant for the DASS‐D and DASS‐A. Further, floor effects (Terwee et al. 2007) were pronounced for the DASS‐D and DASS‐A in both mothers and fathers, while for the EPDS, a floor effect was absent in mothers and less pronounced in fathers; they were also less pronounced for the EPDS‐3A in both parent groups. Thus, compared to the EPDS and EPDA‐3A, the DASS‐D and DASS‐A exhibited poorer score interpretability (Terwee et al. 2007) in both parent groups, possibly affecting their capacity to detect a minimal important difference (Prinsen et al. 2018).

Like the EPDS, DASS‐D discriminant validity for the fathers was exhibited against the anxiety measurements but not the stress measure (DASS‐S). When scrutinizing the DASS‐S items, it was noted that several items (e.g., ‘I tended to overreact to situations’, ‘I found myself getting agitated’ and ‘I was intolerant of anything that kept me from getting on with what I was doing’) appear to reflect an agitation dimension. This may support the notion that feelings of anger are prominent in paternal postnatal depression (Psouni et al. 2017; Macdonald et al. 2020). The typical exclusion of an anger dimension from male depression measurements could warrant further development (Berg et al. 2022; Philpott et al. 2020). Thus, the moderately strong convergent validity herein between the DASS‐D and EPDS in fathers may reflect the lack of male gender‐specific items in both the DASS‐D and EPDS, potentially impacting the construct validity of both these instruments.

The DASS‐A internal consistency was good. The results indicated unsatisfactory convergent and discriminant validities in both parent groups. However, these results seem consistent with results reported from studies of the DASS‐21 in non‐perinatal adult population samples. For example, the current correlations between the DASS‐A and EPDS‐3A were mostly in the low‐to‐moderate range, comparable to the correlation between the DASS‐A and its comparator instrument included in a Swedish study demonstrating DASS‐21 validity (Alfonsson et al. 2017). Furthermore, the finding that some of the DASS‐A correlations with the two depressive symptoms measurements were moderately strong agrees with the well‐known comorbidity between depression and anxiety (Antony et al. 1998; Sanderson et al. 1990).

These findings also line up with the increasing support from confirmatory factor analytic studies for the original three‐factor structure of the DASS‐21 together with a general factor of the total DASS‐21 (i.e., a bifactor structure), while one‐ and two‐factor structures of the DASS‐21 have received insufficient support (Lee et al. 2019; Alfonsson et al. 2017). The cross‐correlations demonstrated in the current study would therefore be in accordance with previous studies which conclude that the DASS‐21 can be used to measure symptoms of depression, anxiety and stress as separate constructs, as well as overall psychological distress using the total DASS‐21 (Lee et al. 2019; Alfonsson et al. 2017; Sinclair et al. 2012).

Regarding previous DASS‐21 results from perinatal maternal populations, one study (Silva et al. 2022) found moderate convergent DASS‐A validity in prenatal women, but did not report discriminant validity against the stress and depression measurements included in the study. Several studies have noted the lack of valid measurement instruments for perinatal anxiety assessment or screening (Sweeney and Wilson 2023; Meades and Ayers 2011; Daire et al. 2022). Considering that the validity of the EPDS‐3A for perinatal screening is yet to be determined (Fairbrother et al. 2019; Loyal et al. 2020; Smith‐Nielsen et al. 2021), and that the existence of the EPDS‐3A has not been supported in fathers (Matthey 2008; Loscalzo et al. 2015; Massoudi et al. 2013), the unsatisfactory convergent validity of the DASS‐A against the EPDS‐3A in both mothers and fathers may be due to low construct validity of one or both anxiety measures. Therefore, further validation of the DASS‐A as a measure of anxiety in perinatal populations is warranted, preferably by including assessments of criterion validity against a gold standard for the diagnosis of anxiety disorders.

Among the three DASS‐21 scales, only the DASS‐S significantly distinguished between maternal and paternal distress levels. This finding is together with the less pronounced floor effects, as compared to the other two DASS‐21 subscales, indicative of better score interpretability of the DASS‐S. The DASS‐S construct validity was not tested against a comparator instrument for the assessment of stress. The DASS‐21 study of pregnant women (Silva et al. 2022) described above found strong DASS‐S convergent validity, whereas DASS‐S correlations with the depression and anxiety measurements included in the study were unreported.

In the current study, correlations between the DASS‐S and the depression measures were moderate or strong in both mothers and fathers. Some correlations with the anxiety measures were moderate. These results are in line with those from the Swedish DASS‐21 study (Alfonsson et al. 2017) analysed in non‐perinatal adult populations, in which the DASS‐S correlated more strongly with DASS‐D than with DASS‐A. They are also consistent with previous results of the DASS‐21 studied in clinical and community populations, demonstrating the highest stress levels in the depressed group, and high also in groups diagnosed with an anxiety disorder (Antony et al. 1998). Furthermore, in the current study, the total DASS‐21 score correlated more strongly with the DASS‐S than with any of the included depression or anxiety measures. This finding is seemingly coherent with the notion that the stress construct as measured by the DASS‐S is a broader construct, closer to general distress than the depressive symptoms and anxiety constructs (Alfonsson et al. 2017). The DASS‐S thus exhibits promising features, although further validation in perinatal parents would be valuable.

### 4.1 Strengths and Limitations

The repeated measures design with three separate timepoints, covering validation of the DASS‐21 in early, middle and late postnatal periods allows stronger interpretation compared to a single timepoint design. Strengths were also the inclusion of fathers besides mothers, as well as several comparison measures allowing assessments of convergent and discriminant validity. The demonstration of measurement properties of the comparator instruments (EPDS and EPDS‐3A) as recommended by COSMIN (Mokkink et al. 2010a) was another strength of the study. A study limitation was the relatively small sample sizes, although null findings were within a context of other strongly significant results and thus improved confidence in interpretations when the null hypothesis was retained.

Structural validity was not assessed due to the COSMIN sample size requirements for factor analysis (Terwee et al. 2007). Another limitation was that the comparator instrument for the DASS‐A, i.e., EPDS‐3A, was insufficiently validated for measuring anxiety, and particularly in fathers. Further, the EPDS‐3A contains only three items which may have contributed to lower correlation coefficients and inconsistent correlation patterns, thus preventing a more conclusive interpretation of the results concerning the DASS‐A.

## 5 Conclusions

In this community postnatal parental population sample, the findings were in line with those previously demonstrated for non‐perinatal adult populations. The results thus indicate that the DASS‐21 could be used during the early, middle and late postnatal periods for assessing parental depressive symptoms, anxiety and stress, as well as general psychological distress. Further analysis in larger perinatal parental samples is needed to confirm this. The DASS‐D exhibited good measurement properties for depressive symptoms in mothers and acceptable properties in fathers, consistent with previous findings in perinatal populations including those from studies using the EPDS. The DASS‐A measurement properties were consistent with previous findings for perinatal anxiety measurement instruments. Further validation of the DASS‐A is therefore warranted. The DASS‐S properties were satisfactory in both parent groups. Altogether, the DASS‐D and DASS‐A seem comparable to other instruments currently used for assessing parental depressive symptoms and anxiety in the perinatal period, while using the DASS‐21 for measuring types of parental distress adds value through its inclusion of a stress measure.

## Author Contributions

**Birgitta Kerstis:** data curation, writing – original draft, writing – review and editing, formal analysis. **Peter Jönsson:** formal analysis, writing – review and editing. **Mariette Derwig:** writing – review and editing. **Kent W. Nilsson:** writing – review and editing. **Inger Kristensson Hallström:** funding acquisition, writing – review and editing. **Sara Lindeberg:** conceptualization, funding acquisition, investigation, data curation, formal analysis, writing – original draft, writing – review and editing, project administration.

## Ethics Statement

The study was approved by the Swedish Central Ethics Committee (Dnr. 2016/587) and conducted according to the Helsinki Declaration (World Medical Association 2002). All participants provided informed consent.

## Conflicts of Interest

The authors declare no conflicts of interest.

## Data Availability

Data sharing is not applicable to this article as no datasets were generated or analysed during the current study.
